# Blue-Enriched Light Enhances Alertness but Impairs Accurate Performance in Evening Chronotypes Driving in the Morning

**DOI:** 10.3389/fpsyg.2018.00688

**Published:** 2018-05-15

**Authors:** Beatriz Rodríguez-Morilla, Juan A. Madrid, Enrique Molina, José Pérez-Navarro, Ángel Correa

**Affiliations:** ^1^Centro de Investigación Mente, Cerebro y Comportamiento, University of Granada, Granada, Spain; ^2^Chronobiology Laboratory, University of Murcia, Murcia, Spain; ^3^Basque Center on Cognition, Brain and Language, San Sebastián, Spain

**Keywords:** chronotype, time of day, driving, alertness, fatigue, accuracy, neuroergonomics, countermeasures

## Abstract

Attention maintenance is highly demanding and typically leads to vigilance decrement along time on task. Therefore, performance in tasks involving vigilance maintenance for long periods, such as driving, tends to deteriorate over time. Cognitive performance has been demonstrated to fluctuate over 24 h of the day (known as circadian oscillations), thus showing peaks and troughs depending on the time of day (leading to optimal and suboptimal times of day, respectively). Consequently, vigilance decrements are more pronounced along time on task when it is performed at suboptimal times of day. According to research, light exposure (especially blue-enriched white) enhances alertness. Thus, it has been proposed to prevent the vigilance decrement under such adverse circumstances. We aimed to explore the effects of blue-enriched white light (vs. dim light) on the performance of a simulated driving task at a suboptimal time of day. A group of evening-types was tested at 8 am, as this chronotype had previously shown their largest vigilance decrement at that time. In the dim light condition, vigilance decrements were expected on both subjective (as increments in the Karolinska Sleepiness Scale scores) and behavioral measures [as slower reaction times (RTs) in the auditory Psychomotor Vigilance Task, slower RTs to unexpected events during driving, and deteriorated driving accuracy along time on task]. Physiological activation was expected to decrease (as indexed by an increase of the distal-proximal temperature gradient, DPG). Under blue-enriched white light, all these trends should be attenuated. Results from the control dim light condition replicated the vigilance decrement in all measures. Most important, the blue-enriched white light attenuated this decrement, leading to both lower DPG and faster RTs. However, it impaired accuracy of driving performance, and did not have any effect on subjective sleepiness. We conclude that exposure to blue-enriched light provides an effective countermeasure to enhance vigilance performance at suboptimal times of day, according to measures such as RTs. However, it should be considered that alerting effects of light could impair accuracy in precision tasks as keeping a proper car position. The current findings provide ergonomic implications for safety and fatigue related management systems.

## Introduction

There is broad evidence that cognitive performance is affected by circadian rhythms (natural oscillations of biological variables with a periodicity of around 24 h; reviewed by Blatter and Cajochen, 2007; Schmidt et al., 2007). Such fluctuations also vary with *chronotype*, i.e., individual preferences regarding sleep-wake timing, which expresses internal differences in the phase of the circadian cycle (Adan et al., 2012). The chronotype can be understood as a continuum, ranging from extreme morning-types (people who show their best cognitive performance early in the morning) to extreme evening-types (performing their best late in the evening or during the night), and vice-versa. Approximately 60% of the population tends to be in the middle of the continuum, corresponding to intermediate chronotypes.

The interaction between time of day and chronotype, known as “synchrony effect” has been reported in many studies employing different cognitive tasks (Yoon, 1997; May and Hasher, 1998; Intons-Peterson et al., 1999; Winocur and Hasher, 2002; Lara et al., 2014), although tasks engaging high operational load or motivation levels may be unaffected by this effect, as reported in Natale et al. (2003) for reasoning tasks. Driving performance, our topic of interest, was sensible to synchrony effects in previous research (Oginska et al., 2010; Correa et al., 2014).

Since driving requires maintenance of attention for long periods, driving performance typically deteriorates along time on task, following the so-called *vigilance decrement*. The vigilance decrement is most evident when driving takes place under adverse circumstances (Folkard, 1997; Di Milia et al., 2011; Correa et al., 2014). For example, epidemiological research (Folkard, 1997; Di Milia et al., 2011) confirms that traffic accidents are most frequent at the circadian trough of arousal promotion (3–5 am). In our previous research (Correa et al., 2014), we found lower performance in evening-type participants driving at early morning (8 am) in comparison to the evening. However, it is not always feasible to schedule tasks at optimal times of day. Therefore, the current research addressed whether this behavioral impairment could be prevented by exposure to short wavelength (blue color) light, given its acute alerting effects on the nervous system (see Cajochen, 2007, for a review). The main practical implication of this investigation is that, testing the effectiveness of non-pharmacological countermeasures to improve alertness in the 20% of the population-related to a higher risk of fatigue-related driving accidents (Di Milia et al., 2011)-, represents a valid research approach in the context of safety and fatigue-related management systems, in the field of transport and ergonomics (Phillips et al., 2017).

Early studies on the alerting effects of light focused on blue light at night, as it is related to melatonin suppression (Cajochen et al., 2005; Lockley et al., 2006). Melatonin secretion is modulated by photic information, so that this hormone is maximally secreted during the dark phase, inducing sleep in humans, and inhibited by light stimulation, which contributes to promote a wake-state during daytime (Moore, 1995). The main photoreceptors involved in this non-visual effect of light are the ipRGCs (intrinsically photosensitive retinal ganglion cells) whose photopigment, melanopsin, is maximally sensitive to light around 460–490 nm (Bailes and Lucas, 2013). Consequently, blue light has shown alerting effects at night at physiological (thermoregulation, heart rate, melatonin secretion; Cajochen et al., 2005), subjective (Karolinska Sleepiness Scale; Cajochen et al., 2005; Chellappa et al., 2011), and behavioral levels [Psychomotor Vigilance Task (PVT); Chellappa et al., 2011].

Regarding driving, studies on the effects of light exposure are very scarce and have not yet provided consistent results. For example, in the study of Taillard et al. (2012), driving performance during nighttime (at 1:00 h and at 3:15 h, for 2 consecutive hours) was improved by exposure to blue light at low-moderate intensities (20 lux, 7.4 μW/cm^2^). By contrast, Phipps-Nelson et al. (2009), did not find benefits from blue-enriched light on night driving (2 h continuously, four times from 21:00 h to 8:00 h), in spite of increasing alertness. Such alerting effect was indexed by EEG slow wave delta and theta suppression, in comparison to an orange light and a control dim light condition. The absence of effects on driving performance was attributed to the low intensity of the light stimulus (1.12 to 1.18 lux, 2 μW/cm^2^).

In line with Phipps-Nelson et al.’s study, we recently found exposure to blue-enriched white light at early night to increase physiological alertness (indexed by decrement of the distal-proximal temperature gradient along the time on task) with no concomitant benefits on driving performance. Rather, driving performance in this condition deteriorated along time of light exposure (Rodríguez-Morilla et al., 2017). It is possible that the increase of physiological alertness could have gone beyond the optimal level of activation required for proper performance in this task, in line with the Yerkes-Dodson’s law (Yerkes and Dodson, 1908). Thus, we were interested on testing the effects of light on driving under more adverse conditions than in our previous study, aiming to optimize the benefits derived from the acute alerting effects of light.

To study light effects under suboptimal circumstances, different designs have been employed besides nighttime testing, such as sleep restriction (Phipps-Nelson et al., 2003; Gabel et al., 2013, 2015) or generating fatigue by mental exertion (Smolders and de Kort, 2014; Borragán et al., 2017). Testing extreme chronotypes at their suboptimal time of day could provide similar conditions of low performance, likely to benefit from possible light effects. But, to our knowledge, this approach has yet to be addressed. Generally, in lighting research on humans, participant’s chronotype is either not considered or controlled by testing intermediate chronotype subjects only. As the closest to our approach, the study of Maierova et al. (2016) evaluated the effects of a bright light (vs. a control condition of dim light) on extreme chronotypes throughout their biological waking period, finding light to improve performance in visual and auditory 3- and 2-back tasks, while simpler tasks as 0-back and PVT task did not benefit from light exposure. But importantly, in Maierova’s study participants were tested according to their biological instead of clock time, so that the first testing time was 7:16 h for morning-types and 11:14 h for evening-types. Therefore, evening-types remained to be tested at early morning clock-times, when they could be expected to show their lowest performance (May and Hasher, 1998; Goldstein et al., 2007; Correa et al., 2014; Lara et al., 2014).

As an alternative approach to that employed by Maierova et al. (2016) (which adapted test timing to preferred sleep-wake timing), we considered also relevant to test evening-types at early morning according to clock-time (e.g., 8:00 h), due to several reasons. First, the evening chronotype is highly prevalent among adolescents and young population (Roenneberg et al., 2007), who are most frequently required to perform at early morning clock-time at school or work. This comes into conflict with the biological time of evening-types, leading to a condition of “chronic jetlag” (Wittmann et al., 2006). As a matter of fact, in evening-types whose midsleep time ranges from 5 to 6 am on forward (Roenneberg et al., 2007) early morning clock-times commonly correspond to the second half of their biological night. Second, in line with the former, our design (i.e., testing evening-types at early morning) guaranteed participants to be under unfavorable circumstances while allowing us to carry out the experiment at daytime, and without the complexity of a sleep deprivation design. Third, as previously mentioned, the effects of light exposure are still to be explored on evening-types at early morning (clock-time).

Considering the above, we studied for the first time the effect of blue-enriched white light (as compared to dim light) over driving performance in a group of evening-types at 8:00 h.

An important implication of this approach is that, in real life, evening-types in the early morning are necessarily affected by partial sleep deprivation, due to externally imposed schedules (Roenneberg et al., 2003). As this inherent reality was the focus of our interest, we had to assume a certain degree of sleep deprivation in our sample. Thus, great care was taken that the total amount of sleep was similar across our different light conditions, in order to control for possible confounds.

In the dim light (control) condition we expected to replicate our previous finding that driving performance at such suboptimal time of day would decrease along time on task (Correa et al., 2014). This decrement should further manifest as higher reaction times (RTs) in the PVT (Dinges et al., 1997), slower reactions and higher deviations of the car position while driving (Correa et al., 2014) and increasing gradient between distal and proximal skin temperatures (Rodríguez-Morilla et al., 2017). In contrast, exposure to blue-enriched white light should attenuate the vigilance decrement, mitigating the increment of RTs, position error and temperature gradient.

## Materials and Methods

### Participants

Seventeen *evening-type* students from the University of Granada with normal or corrected vision participated in this study (ages ranging 19 – 24 years; *M* = 20.25; *SD* = 1.48; 11 women). The study was conducted at Granada, Spain (37°11′17′′N, 03°36′24′′W; UT+02:00) between April, the 4th and June, the 21st of 2016. Exclusion criteria were pregnancy, major medical conditions or medication intake on a regular basis, sleep disorders, night or shift work and the realization of any transmeridional travels within the 3 months prior to the experiment. Additionally, two participants were excluded for coming to at least one of the experimental sessions without sleeping the previous night, and one participant for showing a midsleep time earlier than 4 am, indicating an intermediate instead of evening chronotype. Having a driving license was not taken into account for the selection process, since it did not show any relationship with performance in a similar task in a previous study (Correa et al., 2014). This study was approved by the Ethics Committee of the University of Granada (n.34/CEIH/2015). All participants gave prior written informed consent and they were rewarded economically at the end of the experiment.

### Materials and Procedure

#### Circadian Rhythms and Sleep

Circadian rhythms and sleep quality of the participants during the week comprising the experimental sessions were assessed by an ambulatory circadian monitoring equipment (*Kronowise*^®^, Chronolab, University of Murcia), which participants were instructed to wear 24 h per day except for shower time (as described in Ortiz-Tudela et al., 2010). This equipment integrates two different devices: a temperature sensor (Thermochron^®^ iButton DS1921H, Dallas, Maxim) placed on the non-dominant wrist for measuring the distal temperature rhythm every 10 min, and an actimeter (Hobo^®^ Pendant G Acceleration Data Logger) placed on the arm for registering the rhythms of motor activity and body position every 30 s.

Body temperature has shown to be a reliable marker of the circadian status (Kerkhof and Van Dongen, 1996; Sarabia et al., 2008). In particular, while core body temperature drops when an individual is sleeping, peripheral (e.g., wrist) skin temperature starts to increase before bedtime and falls just after awakening (Sarabia et al., 2008). Furthermore, wrist temperature is more closely related than core temperature to sleepiness and sleep onset (Kräuchi et al., 2005), and shows high correlation between the evening increase and the DLMO (Bonmati-Carrion et al., 2014). In fact, body temperature has recently been accepted as an alternative and less invasive method than melatonin assessment to assess circadian phase timing (Mullington et al., 2016). Likewise, actigraphy has been used for around 30 years for assessing circadian and sleep rhythms (Ancoli-Israel et al., 2003).

The combination of actigraphy and wrist temperature recordings yields more accurate assessment of circadian rhythms and estimation of sleep (Ortiz-Tudela et al., 2010, 2014). Indeed, in the latter study, the estimations of sleep latency (min), total sleep time (min), sleep efficiency (%) and number of awakenings obtained from this integrative procedure did not statistically differ from those estimated from PSG.

#### Questionnaires and Subjective Measurements

Participants were interviewed about health and sleep habits, including information related to timing and duration of sleep, subjective sleep quality, and consumption of any stimulating or relaxing substances. These questions were answered in reference to both general habits (as screening information) and the nights prior to each experimental session.

*Morningness – Eveningness Questionnaire* (*MEQ*) (Horne and Östberg, 1976), in its Spanish reduced version (Adan and Almirall, 1990): scores in this questionnaire can range between 4 and 25, leading to three groups: *evening type* (score 4–11), *neither type* (12–17), *morning type* (18–25). We employed this questionnaire as a screening measure to select exclusively evening-type participants.

*Karolinska Sleepiness Scale* (*KSS*) (Åkerstedt and Gillberg, 1990): it measured the subjective sleepiness level at both the beginning and end of the experimental sessions, ranging from 1 = “totally alert” to 9 = “totally sleepy, difficulties to keep on awake.” It was administered via computer screen.

Mood state scale: participants reported about their general mood state from 1 = “extremely negative” to 9 = “extremely positive” at both the beginning and end of the experimental sessions, via computer screen.

#### Behavioral Tasks

##### Psychomotor vigilance task (PVT)

We used an auditory version of this computerized reaction-time (RT) task that evaluates sustained attention (Dinges and Powell, 1985), programmed through E-Prime software (Schneider et al., 2002). In the current version, the target stimulus was a 700-Hz tone of 500 ms long presented after a delay interval ranging randomly on each trial from 2000 to 10000 ms. The participants had to respond to the target stimulus as quickly as possible by pressing a key. In every trial, the RT was recorded as dependent variable and displayed to the participants as feedback for 500 ms, and then the next trial began. Participants also received feedback on misses (responses after 1500 ms) and anticipations (responses before target onset). This task was presented for 10 min without interruption.

##### Driving simulator

**FIGURE 1:**
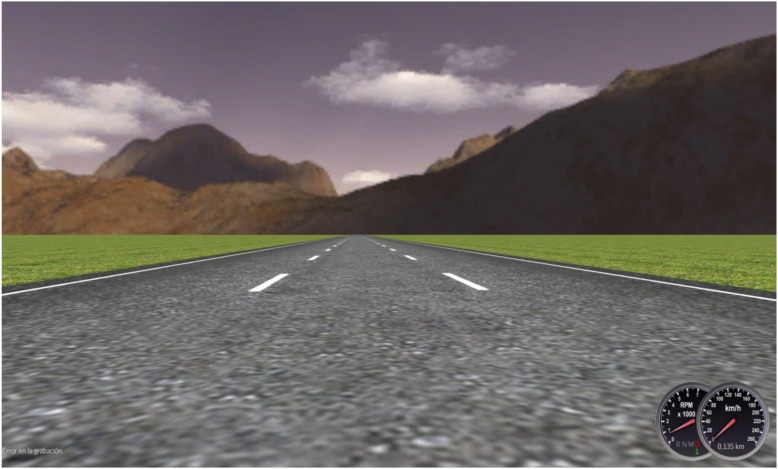
Simulated driving task display. Participants were asked to drive the car as centered as possible along the central lane.

The software employed for simulated driving was OpenDS 2.2 (Sun Microsystems©). The car was controlled through a Logitec Momo Racing wheel and pedals set. The participants were instructed to drive the car as centered as possible along the central lane of a straight highway with three lanes (**Figure 1**).

After a delay randomly ranging from 6000 to 14000 ms, the car was displaced from its position, simulating being pushed away by a gust of wind. The participants’ task was to correct the car back to the central position as quickly as possible. Two main dependent variables were recorded: RT to correct the position of the car after the wind events, and position error of the car (i.e., the distance with respect to the center of the lane), which was continuously recorded. The recording of RT constituted an advantage with respect to the software employed in our previous study (Rodríguez-Morilla et al., 2017) due to its sensitivity to vigilance fluctuations and its relevance to safety.

As we intended to simulate real highway driving, i.e., keeping a proper position of the car and responding quickly to unexpected events during driving, the task was deliberately presented in the visual modality, similarly to previous research (Phipps-Nelson et al., 2009). Visual stimuli were displayed on a 24′ LCD monitor, rating 100 – 240 V∼, 50/60 Hz.

#### Physiological Measurements During Sessions

Additional temperature sensors (Thermochron^®^ iButton DS1921H, Dallas, Maxim) assessed distal (right wrist) and proximal (right infraclavicular area and the inner side of the upper arm – peri-axilar –) skin temperature every minute throughout the session, as objective markers of physiological alertness (Kräuchi et al., 2005; Sarabia et al., 2008). Although infra-clavicular recording is widely established as proximal temperature (Hasselberg et al., 2013), we added the inner arm temperature (Wuyts et al., 2012) since it corresponds to a more proximal body region and, therefore, was expected to yield closer estimations of central temperature.

#### Light Manipulation

**FIGURE 2:**
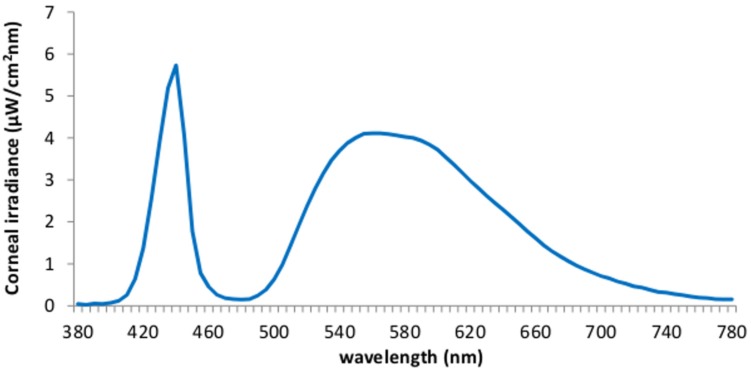
spectral distribution of the blue-enriched light at the corneal level.

We employed a 40 w LED lamp (IgniaLight, SACOPA, S.A.U.) emitting blue-enriched polychromatic white light with maximum peak of spectral irradiance at 440 nm and illuminance at the eyes level, 469 lux. The spectral distribution and irradiance were selected in line with our previous study (Rodríguez-Morilla et al., 2017), intending to maximize alerting effects. The spectral composition of the lighting source, determined by means of an Illuminance Spectrophotometer (Konica Minolta CL-500A), is displayed on **Figure 2**.

The lamp was placed obliquely from the left side, 60 cm far from the participants’ eyes, so indirectly falling upon both participants’ eyes and the screen, and the light emitted was diffused by a shade, mimicking a Ganzfeld full-field illumination. Further photometric information is reported in **Table 1**. This condition was compared to a dim light control condition (lights off in the same room, <1 lux).

**Table 1:** photometric values of the light source at eye level, estimated through the Excel toolbox from Lucas et al. (2014).

Irradiance (μW/cm^2^)	141.14
Peak of spectral irradiance	440 nm
Photon flux (1/cm^2^/s)	4^∗^10^14^
Photopic illuminance (lux)	469
Cyanopic	323.26
Melanopic	224.84
Rhodopic	294.64
Chloropic	401.00
Erythropic	444.34

Illuminance emitted by the monitor was 0.06 lux, which is below the threshold required for causing alerting effects on the nervous system (Cajochen et al., 2000; Wood et al., 2013). In addition, the blue component of the light emitted by this monitor was filtered by F-lux^®^ software.

### Study Protocol

Only evening-type volunteers, i.e., with rMEQ scores below 12, and later confirmed by the phase markers calculated from ambulatory circadian monitoring, were included. Every participant came three times to the laboratory. The first time, they were given the circadian monitoring devices to assess their circadian rhythms under normal living conditions during 1 week. They were instructed to follow regular sleep-wake schedules following their own internal preferences (i.e., their usual schedules at both working and free days), as we were interested on realistic consequences of performing at an adverse time of day considering chronotype and externally imposed schedules. In any case, participants were asked to keep similar sleep duration and timing between the nights prior to each experimental session. Within this week, every participant completed two experimental sessions at non-consecutive days, following a within-subject design in which blue-enriched and dim light conditions were administered in a counterbalanced order.

**FIGURE 3:**
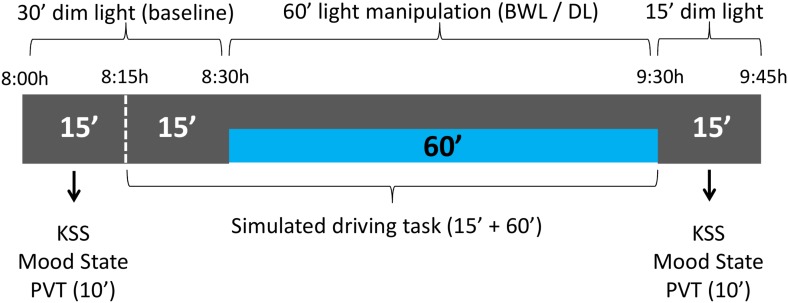
schema of the experimental protocol. The participants remained the first 30 min of the session under dim light, while they carried out the baseline subjective scales and PVT, followed by 5 min of driving practice and 10 min of baseline driving. After that, one of the lighting conditions (blue-enriched white – BWL – or dim light – DL –) was applied while they drove for 60 min. Finally, they performed the PVT and subjective scales for a second time in dim light.

Each session lasted about 105 min and started at 8 am. Participants were seated at 60 cm from the monitor in a soundproof isolated room throughout the whole session. During the first 30 min, the participants were under dim light conditions in order to allow dark adaptation (Fain et al., 2001) and to obtain temperature and performance baselines (see **Figure 3**). Along this period, they completed the mood state and sleepiness (KSS) scales, performed the PVT and practiced the simulated driving task for 5 min, followed by 10 min of driving baseline performance. Then, the blue-enriched white light (or no light, in the dim light condition) was applied, and the participants performed the driving task for another 60 min. After driving, they were asked again about their level of sleepiness and mood state, and performed the PVT for a second time.

Caffeine consumption was allowed the day of the experimental session (but not during the session itself) in line with participants’ habits. However, they were asked to behave similarly across conditions (i.e., those who consumed caffeine the day of the first experimental session as habitual consumers, were asked to also consume it, in a similar amount, the day of the second experimental session). This information was recorded, and the distribution of consumption across experimental conditions was analyzed in order to control for possible confounds.

### Design and Data Analysis

#### Circadian Rhythms and Sleep

The rhythms of motor activity, body position, wrist temperature, and environmental light were processed as described in previous studies (Ortiz-Tudela et al., 2010). An integrated variable “TAP” (from Temperature, Activity and Position) was obtained from the rhythms of wrist temperature, motor activity and body position to infer the general level of activation and sleep-wake states. Values of the three variables were normalized in a scale ranging from 0 to 1, and temperatures were then subtracted from 1 to obtain its inverse rhythm. Then, the three variables were averaged, obtaining a new value ranging from 0 to 1. Thereby, low values of this new integrated variable expressed low levels of body position (i.e., near horizontal positions, obtained when the subject is lying out), scarce movements and high wrist temperatures, associated with low activation or sleep (Sarabia et al., 2008; Krauchi and Deboer, 2010; further details on sleep estimation from TAP in Ortiz-Tudela et al. (2010, 2014). The TAP algorithm has shown to estimate sleep more accurately than any of the single variables alone, both in comparison with sleep diaries (Ortiz-Tudela et al., 2010) and PSG (Ortiz-Tudela et al., 2014).

**FIGURE 4:**
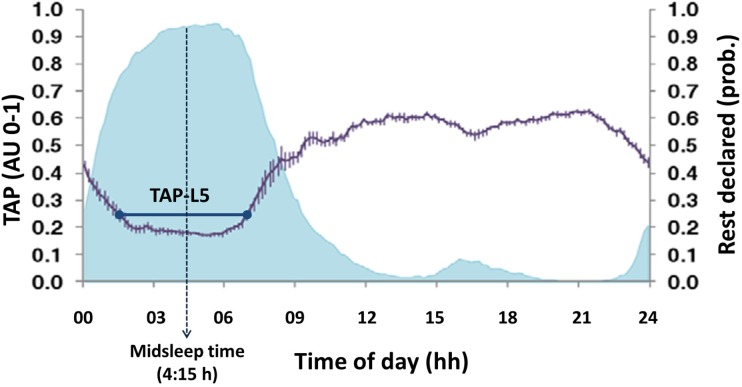
Example of mean circadian rhythm of TAP (modified from Ortiz-Tudela et al., 2010). The violet line represents the mean waveform of TAP rhythm (value ± SEM, in arbitrary units from 0 to 1) obtained from the week recordings of wrist temperature, motor activity and body position of a sample of 49 subjects. The shaded blue area corresponds to sleep declared by subjects. The five consecutive hours of lowest values (L5) are marked by the horizontal dark blue line. The central time of that period (4:15 h approximately) is considered as midsleep time.

Each of these variables, including TAP, was then submitted to non-parametric analyses (Refinetti et al., 2007), providing the following estimators of sleep phase:

M5/L5: mean values of every variable during the five consecutive hours where skin temperature was maximal (M5) and the values of motor activity, body position and TAP were the lowest (L5). This period was identified as the main rest period (**Figure 4**).

Midsleep time: central time of the sleep period, located from the central time of L5 from TAP. It was employed as a physiological index of the sleep phase and, consequently, an objective measure of chronotype (see **Figure 4**).

#### Behavioral and Temperature Measures

Driving performance was analyzed following a repeated-measures design with the factors *light condition* (blue-enriched versus dim light) and *time on task* (20, 40, and 60 min after starting the light manipulation period), as in our previous study (Rodríguez-Morilla et al., 2017). Planned comparisons between light conditions on each level of time on task were performed in order to study the temporal course of light effects on performance (see Vandewalle and Dijk, 2013; Rodríguez-Morilla et al., 2017; Smolders and de Kort, 2017, for analogous approaches). The dependent variables were mean RT and position error per minute, baseline corrected.

Two estimations of the distal-proximal temperature gradient (DPG) were conducted for every participant by subtracting infra-clavicular (DPG-clavicle) and inner arm temperature (DPG-arm), respectively, from the values of the wrist temperature. The dependent variable was the temperature change with respect to the last minute of the dim light phase, when temperatures were maximally stabilized. Changes in both DPG-clavicle and DPG-arm were analyzed following a similar 2 (*light condition*) × 3 (*time on task*) design.

The analysis of PVT performance included mean RTs longer than 100 ms, and followed a repeated measures design with the factors *light condition* and *time of testing* (pre- and post- driving task).

Data were analyzed through a non-parametric permutation test. It consists of performing all possible exchanges of conditions’ labels from the original sample, assuming that, given the null hypothesis of no differences between conditions, this replacement would not change the probability distribution of the samples. This generates an empirical virtual population, making this procedure independent from the underlying data distribution and adequate for small sample sizes (Pesarin and Salmaso, 2010). We obtained a distribution of 10,000 permutations, and *p*-value was calculated as the proportion of values from this surrogate distribution which were higher than the value obtained from the original sample. All these analyses were carried out through Matlab^®^ (Mathworks^®^), following the algorithms described by Good (2005). Non-parametric bootstrap methodology was used to calculate effect sizes of the significant effects obtained by permutation analyses.

Since demographic and subjective data had single observations per level, squared-chi tests were employed to compare the frequency distributions of caffeine intake before each session, and sleep duration in the night prior to each session was analyzed through a one-way analysis of variance (ANOVA) with the factor of *light condition*. Subjective measures of sleepiness (KSS) and mood were analyzed through 2 (*light condition)* by 2 (*time of testing*: pre- and post- driving task) ANOVAs. Effect sizes of significant effects obtained through ANOVAs were estimated and reported as partial eta squared ($\eta_{p}^{2}$).

## Results

### Circadian Rhythms, Demographic Data and Questionnaires

**FIGURE 5:**
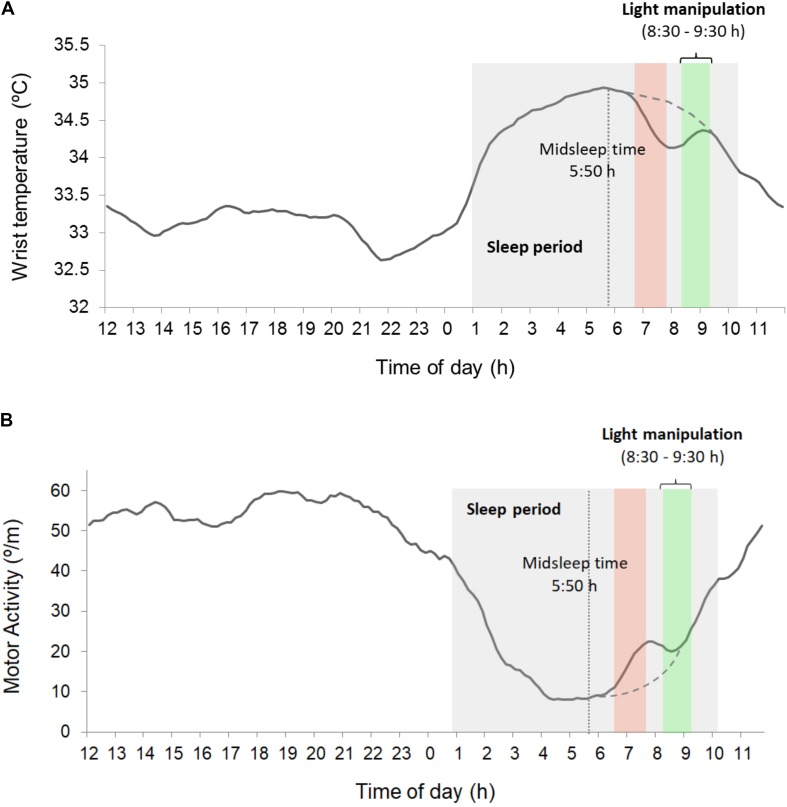
**(A)** Mean temperature circadian wave. **(B)** Mean motor activity wave. The mean sleep period, estimated from TAP (Ortiz-Tudela et al., 2010, 2014) and characterized by the highest temperature values and lowest motor activity values, is shaded in gray. The temperature drop and activity rise between approximately 7:00 h and 8:00 h (red bar) correspond to the wakeups and displacements to the laboratory on experimental session days. The dashed line represents the expected temperature and motor activity patterns, respectively, at that time of day (obtained from free days). The green bar indicates the light exposure period during the experimental session.

Both the rMEQ score (*M* = 9.21, see **Table 2**) and midsleep time (05:50 h) confirmed the *evening-type* chronotype of our sample. **Figures 5A,B** represent the circadian rhythms of wrist temperature and motor activity, respectively, averaged across participants during the week including the experimental sessions. Inspection of these figures shows both a temperature drop and an activity rise matching the timing of the laboratory sessions, soon after the midsleep time. Thus, the period characterized by high distal temperature and low motor activity typically associated to morning sleep in evening-types was presumably interrupted by their attendance to the laboratory. This confirms that the experimental sessions took place during a circadian time naturally linked to sleep in our sample.

**Table 2:** mean values and standard deviations (in parenthesis) of demographic data, and mean scores and 95% confidence intervals [C.I., in square brackets] obtained through bootstrapping of subjective measures (*n* = 17).

Age	20.25 (1.48)	
Chronotype (rMEQ)	9.21 (1.65)	
Midsleep time (hh:mm)	5:50 h (1:17)	

	**Blue-enriched white light**	**Dim light**

Sleep hours (before experimental sessions)	5.68 (1.06)	5.27 (0.97)
Sleep onset (before experimental sessions)	1:36 h (1:27)	1:32 h (1:13)
Wake time (before experimental sessions)	6:50 h (0:29)	6:52 h (0:35)
Subjective sleepiness (KSS)		
Pre	4.69 [3.94 – 5.5]	5.63 [4.69 – 6.44]
Post	6.13 [5.31 – 6.81]	6.69 [6 – 7.38]
Subjective mood state (1 = extremely bad; 9 = extremely good)		
Pre	6.94 [6.31 – 7.5]	6.75 [5.88 – 7.31]
Post	6.87 [6.38 – 7.38]	6.63 [5.88 – 7.19]

There were not significant differences in the total amount of sleep between nights prior to each experimental session [5.68 h for the *light exposure* condition versus 5.27 h before the *dim light* session: *F*(1,16) = 2.88, *p* = 0.11], or in the distribution of caffeine intake before the experiment across sessions (33% of participants consuming caffeine in blue-enriched white light and 40% in dim light, χ^2^_1_ = 0.144; *p* = 0.705).

The analysis of subjective sleepiness as measured by the KSS showed a significant effect of *time of testing*, *F*(1,15) = 11.54; *p* < 0.01; $\eta_{p}^{2}$ = 0.43, revealing higher subjective somnolence/sleepiness after (mean = 6.44; *SD* = 1.52) than before (mean = 5.16; *SD* = 1.74) the driving task. A significant effect of *light condition, F*(1,15) = 5.09; *p* = 0.04; $\eta_{p}^{2}$ = 0.25, indicated higher levels of sleepiness in the dim light (*M* = 6.16; *SD* = 1.72) than in the blue-enriched light condition (mean = 5.41; *SD* = 1.74), but the *light* × *time of testing* interaction was not significant (*F* < 1), suggesting that differences in subjective sleepiness were previous to light administration. There were no significant effects regarding mood state (all *p*s > 0.24).

### Skin Temperature Measures

All main effects and interactions obtained from permutation analyses on every temperature assessment are reported in **Table 3**.

**Table 3:** Main effects, interactions, and pairwise contrasts in all temperature analyses.

	Wrist	Clavicle	Inner-arm	DPG-clav.	DPG-arm
Light condition	*p* = 0.005	*p* < 0.001	*p* = 0.127	*p* < 0.001	*p* = 0.127
Time on task	*p* < 0.001	*p* < 0.001	*p* = 0.213	*p* < 0.001	*p* < 0.001
Interaction	*p* = 0.001	*p* = 0.026	*p* = 0.188	*p* = 0.001	*p* = 0.001
Time on task in DL^1^	*p* < 0.001	*p* < 0.001	*p* = 0.028	*p* < 0.001	*p* < 0.001
Time on task in BWL^1^	*p* < 0.001	*p* < 0.001	*p* = 0.152	*p* < 0.001	*p* < 0.001
Light effect^2^ 20 min	*p* < 0.001	*p* < 0.001	*p* = 0.528	*p* < 0.001	*p* < 0.001
Light effect^2^ 40 min	*p* = 0.008	*p* < 0.001	*p* = 0.037	*p* < 0.001	*p* = 0.346
Light effect^2^ 60 min	*p* = 0.109	*p* = 0.001	*p* = 0.478	*p* = 0.503	*p* = 0.071

*^1^Main effect of time on task in permutation analyses performed separately for each light condition (DL, dim light; BWL, blue-enriched white light). ^2^Pairwise contrasts of factor light condition for every level of time on task.*

**FIGURE 6:**
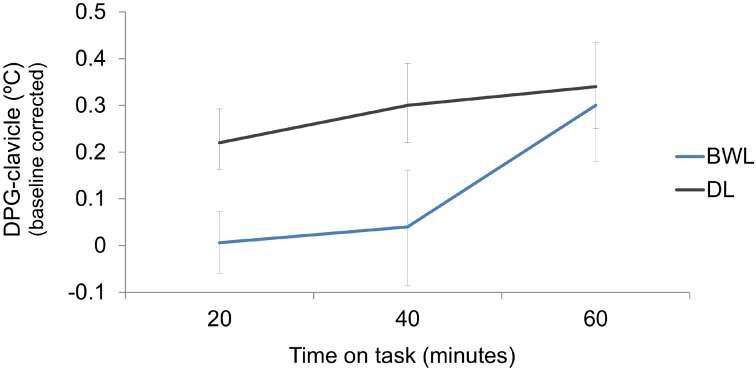
Increments with respect to baseline of the distal-proximal (clavicle) temperature gradient along the driving task, as a function of light condition (BWL, blue-enriched white light; DL, dim light). Bars represent 95% confidence intervals obtained through bootstrapping.

The table shows that all temperature measures, except for the most proximal temperature (inner-arm) showed significant effects of *time on task*, and *time on task* × *light condition* interaction. Wrist, clavicle and distal-proximal (clavicle) gradient also showed a main effect of *light condition*. For comparison purposes with our previous study (Rodríguez-Morilla et al., 2017), description of results will focus on the distal-proximal temperature gradient (**Figure 6**, DPG-clavicle). This temperature gradient significantly increased along *time on task*, but it was reduced by the blue-enriched white light in relation to dim light. Analyses of the significant interaction (*p* = 0.001) revealed that this temperature decrement by blue-enriched white light was already present within the first 20 min and remained significant after 40 min of driving, whereas the lighting effect vanished during the last 20 min of the task.

### Performance in the Psychomotor Vigilance Task (PVT)

**FIGURE 7:**
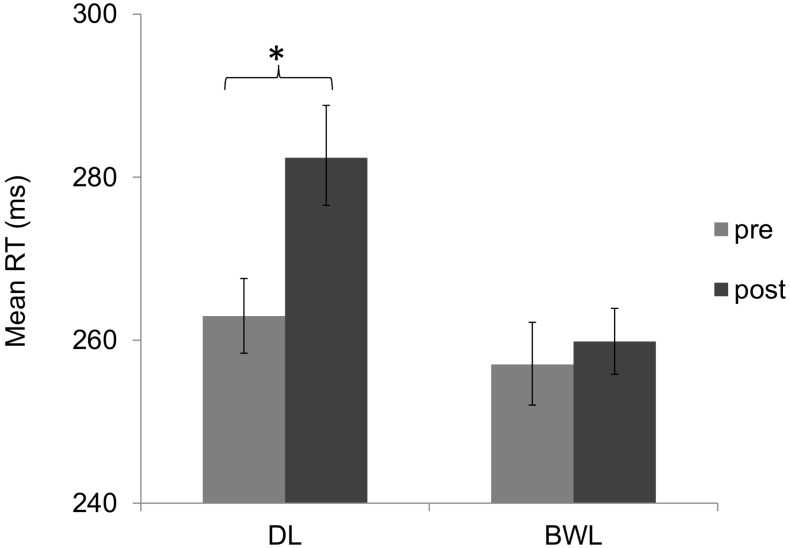
Mean reaction times in the psychomotor vigilance task (PVT) as a function of lighting condition (dim light vs. blue-enriched white light) and time of testing: before (pre) and after (post) the driving task. Bars represent 95% confidence intervals obtained through bootstrapping. ^∗^Significant difference.

The permutation test for the analysis of RTs in the PVT showed a significant effect of *light condition* (*p* < 0.001; 95% CI for the difference of means = 11, 17.74), with faster overall RTs in blue-enriched (*M* = 260 ms; 95% C.I. = 257 – 265) as compared to dim light (*M* = 276 ms; 95% C.I. = 271 – 281), and a significant effect of *time of testing* (*p* < 0.001; 95% CI for the difference of means = 7.87, 14.56), with slower RTs after (*M* = 274 ms; 95% C.I. = 270 – 279) than before (*M* = 262 ms; 95% C.I. = 258 – 266) the driving task. Most relevant, the *light* × *time of testing* interaction, *p* < 0.001, revealed that the RT increment along time was attenuated under blue-enriched (*p* = 0.162) as compared to dim light (*p* < 0.001; 95% CI for the difference of means = 15.08, 24.42) (**Figure 7**).

### Performance in the Simulated Driving Task

**FIGURE 8:**
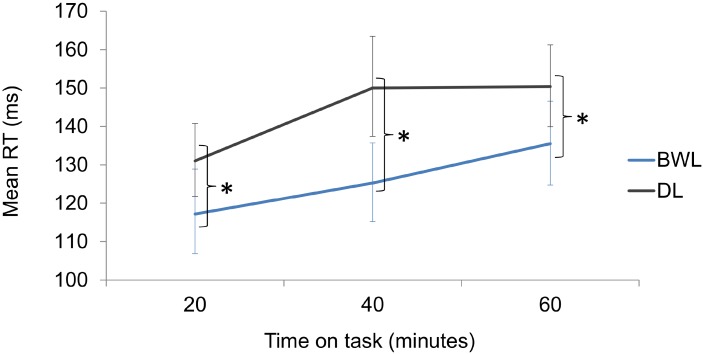
Reaction time increments from baseline in the driving task as a function of time on task and lighting condition (BWL, blue-enriched white light; DL, dim light). Bars represent 95% C.I. obtained through bootstrapping. ^∗^Significant difference.

The permutation analysis on driving RTs yielded a significant effect of *light condition* (*p* < 0.001; 95% for the difference of means C.I. = 10.36, 25.2), with faster responses in blue-enriched white (increment from baseline: *M* = 126 ms; 95% C.I. = 120 – 132) vs. dim light condition (*M* = 144 ms; 95% C.I. = 138 – 141). A significant effect of *time on task* (*p* < 0.001; 95% CI for the difference of means between blocks 1 and 3 = 9.39, 28.1) reflected an increase of RTs along driving. The interaction was not significant (*p* = 0.482), as planned comparisons showed that lighting effects were already present within 20 min of exposure, (*p* = 0.042; 95% for the difference of means C.I. = 2.18, 25.73) and remained significant throughout the task (*p* = 0.001; 95% for the difference of means C.I. = 11.95, 38.28 after 40 min and *p* = 0.031; 95% for the difference of means C.I. = 2.93, 27.21 after 60 min) (**Figure 8**).

**FIGURE 9:**
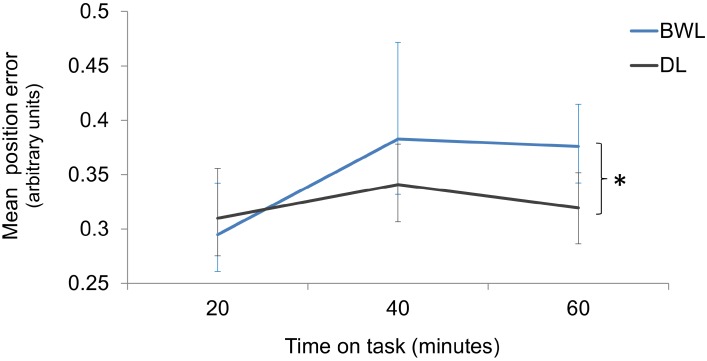
Position error increments from baseline as a function of time on task and lighting condition (BWL, blue-enriched white light; DL, dim light). Bars represent 95% C.I., obtained through bootstrapping. ^∗^Significant difference.

Interestingly, the analysis on driving accuracy revealed higher position error increments with respect to baseline in the blue-enriched white light than in the dim light condition (*p* = 0.002; 95% for the difference of means C.I. = 0.02, 0.08). Position error also increased along *time on task* (*p* = 0.007; 95% C.I. for the difference of means between blocks 1 and 3 = 0.01, 0.08). The *light* × *time on task* interaction was not significant (*p* = 0.188). According to planned comparisons, position error did not differ between conditions within the first 20 min (*p* = 0.816) or after 40 min (*p* = 0.072) of light exposure, but it was significantly larger after 60 min (*p* = 0.001; 95% for the difference of means C.I. = 0.04, 0.12) of blue-enriched white vs. dim light (**Figure 9**).

## Discussion

Exposure to blue-enriched light has been proposed as a resource for enhancing alertness and cognitive performance in adverse circumstances, such as at nighttime (Lockley et al., 2006; Phipps-Nelson et al., 2009; Chellappa et al., 2011; Kretschmer et al., 2011; Taillard et al., 2012; Correa et al., 2016) or after sleep restriction (Phipps-Nelson et al., 2003; Gabel et al., 2013, 2015). Cognitive effects of light have been studied also during daytime (Vandewalle et al., 2006, 2007, 2011; Huiberts et al., 2015; Smolders and de Kort, 2017), but its influence in extreme chronotypes performing at their suboptimal time of day had not been addressed. As we previously reported performance impairments in evening-types driving at early morning (Correa et al., 2014), the current study tested whether blue-enriched white light could attenuate this impairment in another group of evening-types driving at the same time of day (8 in the morning).

We focused in blue-enriched white light as it has shown stronger alerting effects than other kinds of light in our previous study in the evening (Rodríguez-Morilla et al., 2017) and also during daytime (Vandewalle et al., 2007; Rahman et al., 2014). Our design did not allow attributing the possible effects of blue-enriched white light exposure, in comparison to dim light, to the spectral quality of the light employed. Therefore, all the results here showed and discussed were interpreted as effects of bright light exposure and not necessarily due to the blue component of the spectrum.

Our current design involved the following methodological improvements with respect to our previous lighting study (Rodríguez-Morilla et al., 2017): (1) light exposure was manipulated within-subjects to enhance statistical power by minimizing inter-individual differences, (2) the new driving task further measured RTs, as they are sensitive to vigilance fluctuations (Graw et al., 2004; Basner and Dinges, 2011), and (3) non-parametric permutation analyses were performed, being this procedure independent from the underlying data distribution and adequate for a relatively small sample (Pesarin and Salmaso, 2010).

The present study provided two main findings. First, it replicated our previous result (Correa et al., 2014) of a decrement associated with time on task when evening-type participants performed at their suboptimal time of day (8 am). This impairment was clear at multiple dimensions, such as in subjective somnolence (higher KSS scores), physiological (increasing temperatures), and behavioral measures (higher RTs in the PVT, slower responses and higher position error when driving), therefore providing converging evidence of a vigilance decrement. Second, and most important, exposure to blue-enriched white light prevented this decrement, as revealed by lower skin temperatures and faster RTs in both PVT and driving tasks, thus extending the alerting effects of light to a sample of extreme chronotypes. Finally, in line with our previous lighting study (Rodríguez-Morilla et al., 2017), blue light was related to lower accuracy during driving.

Our temperature results showed increments along time on task in all measures except for the most proximal temperature (inner-arm). The inner-arm index could be a more stable estimate of central temperature, not so sensitive to short term variations along time on task. However, the distal-proximal inner-arm gradient was as sensitive as the distal-proximal infraclavicular gradient to measure physiological changes associated with the effects of time on task and lighting. Our finding of increments in distal temperature and distal-proximal gradients have been related to a parasympathetic predominance, as the autonomous nervous system drives vasodilatation on peripheral skin vessels to promote heat loss, diminishing arousal and leading to increased somnolence (Kräuchi et al., 2004, 2005; Sarabia et al., 2008). This finding fits well with the fact that our participants were tested at a suboptimal time of day in a task that typically depletes resources of vigilant attention. Skin temperature increments were also in line with our previous study (Rodríguez-Morilla et al., 2017), where the distal-proximal gradient increased from 22:00 h onwards under dim light. Interestingly, blue-enriched light reduced temperature, indicating a restoration of the level of alertness as compared to the dim light condition (see also Myers and Badia, 1993; Cajochen et al., 2005).

Time on task effects, presumably related to fatigue and decrement in vigilant attention, were also evident at the behavioral level in the PVT. Participants in the dim light condition performed the PVT significantly slower after the driving task than before. Importantly, this RT lengthening was prevented in the blue-enriched light condition, leading to faster performance than in dim light in the PVT administered post-driving. This finding supports previous research showing alerting effects of light on the PVT (Phipps-Nelson et al., 2009; Chellappa et al., 2011), although null results are also common in the literature (Gabel et al., 2015; Huiberts et al., 2016; Segal et al., 2016; Rodríguez-Morilla et al., 2017). Probably, our design optimized sensitivity to such effects by testing participants at suboptimal conditions, as in Phipps-Nelson et al. (2003).

In the simulated driving task, the vigilance decrement was evident as increasing RTs along time on task, in line with our previous finding of clear vigilance decrements in evening-type participants driving at 8 am (Correa et al., 2014). In these conditions, the blue-enriched light helped them to drive with enhanced alertness, as suggested by the finding of faster responses to unexpected events (gusts of wind). Nevertheless, increments in phasic alertness do not always involve performance benefits, rather, it depends on task demands (Yerkes and Dodson, 1908). It has been suggested that phasic alertness improves response speed but at the cost of accuracy (Posner, 1978). Our results concerning position error pointed to this direction. In particular, exposure to blue-enriched white light was associated with a larger increase of position errors from baseline as compared to dim light. This result replicated previous findings of our lab (Rodríguez-Morilla et al., 2017), suggesting that excessive arousal could be detrimental for accurate performance, in line with a speed accuracy trade-off. However, in previous light studies on night driving the position error increased across consecutive testing times, and exposure to blue-enriched light either prevented it (Taillard et al., 2012) or did not increase it with respect to control conditions (Phipps-Nelson et al., 2009). Protocol differences, like shorter task durations or higher illuminance in our study, could account for these differences.

It is also important to note that partial sleep deprivation could have contributed to the effects. Indeed, mean sleep duration was below 6 h in our sample. As we were interested on testing evening-type participants under normal living conditions (e.g., Correa et al., 2014), we assumed a certain degree of sleep deprivation. In real life, evening-types in the early morning are necessarily affected by partial sleep deprivation when there is an obligation of waking up early according to externally (professionally academic) imposed schedules, leading to an accumulation of sleep deficit along working days (Roenneberg et al., 2003). This desynchronization of rhythms between working and free days, which reflects a misalignment between social and biological time, is known as social jetlag. Further, the evening-type rhythm has been compared to a sort of “chronic jetlag” (Wittmann et al., 2006). In any case, our analyses on free-living recording of actigraphy and temperature rhythms, confirmed that sleep duration was balanced across experimental sessions. An alternative approach to avoid sleep deprivation would imply a later testing time, as addressed in Maierova et al. (2016), which tested evening-types from 11:17 h forward, leaving suboptimal morning hours unexplored.

In another vein, our design could have been optimized by either testing our sample also at their optimal time of day (i.e., in the evening), or by comparing it to a sample of morning-types at the same time of day. Therefore, the consideration of our testing time as suboptimal for our sample can only rely on our previous research showing evening-types’ worst performance at early morning (Correa et al., 2014). Indeed, future studies should test larger samples including all chronotypes following a more continuous approach (i.e., testing morning, intermediate and evening-types, scoring along the whole continuum based on both questionnaires and circadian monitoring measures). The additional use of both constant routine and forced-desynchrony protocols should provide further useful information within the framework of sleep and circadian physiology. However, these outstanding issues go beyond the scope of the current research, which rather addressed a well-known problem in our current society: about 20% of the population is evening-type and has to drive under suboptimal circadian and sleep circumstances due to a morning-oriented social schedule, leading to “social jet-lag,” adverse circadian phase and partial sleep deprivation, all of them related to increased risk of fatigue-related driving accidents (Di Milia et al., 2011).

We should note that the spectral composition and intensity of our light source was intended to maximize the alerting effects. However, such light intensities could not be applicable to real life driving. Thus, possible applications of light interventions for improving real driving would require further research using more realistic driving simulators and light sources, so as studying the effects of different light spectra.

## Conclusion

This study addressed for the first time the effects of light exposure on the performance of evening-types driving in the morning. The current contribution in relation to previous research involves a careful selection, on the basis of both subjective self-reports and objective biological markers, of a sample of evening chronotypes to explore the potential benefits of blue-enriched light on performance under this suboptimal circadian and sleep state.

Our results suggest that blue-enriched white light can enhance alertness and prevent fatigue-related behavioral decrements of vigilance at suboptimal times of day, while accuracy in complex tasks requiring precision may be deteriorated. The replication of our previous finding of impaired driving performance under blue lighting (Rodríguez-Morilla et al., 2017) invites careful reflection to the misleading claim that light exposure is a remedy for improving cognitive performance under any circumstance. Hence, a cognitive analysis of tasks involving health risks should be considered when using lighting stimuli to boost alertness. The current research provides implications for ergonomics in the context of safety and fatigue related management systems.

## Ethics Statement

This study was carried out in accordance with the recommendations of the Ethics Committee of the University of Granada with written informed consent from all subjects. All subjects gave written informed consent in accordance with the Declaration of Helsinki. The protocol was approved by the Ethics Committee of the University of Granada (n.34/CEIH/2015).

## Author Contributions

ÁC and JM: conceptualization, funding acquisition, project administration, resources, supervision, and writing – review and editing. BR-M, JM, EM, JP-N, and ÁC: data curation, formal analysis, investigation, and methodology. BR-M and EM: software. BR-M, JM, EM, and ÁC: validation and visualization. BR-M: writing – original draft.

## Conflict of Interest Statement

The authors declare that the research was conducted in the absence of any commercial or financial relationships that could be construed as a potential conflict of interest.
